# Keratins Regulate p38MAPK-Dependent Desmoglein Binding Properties in Pemphigus

**DOI:** 10.3389/fimmu.2018.00528

**Published:** 2018-03-19

**Authors:** Franziska Vielmuth, Elias Walter, Michael Fuchs, Mariya Y. Radeva, Fanny Buechau, Thomas M. Magin, Volker Spindler, Jens Waschke

**Affiliations:** ^1^Faculty of Medicine, Institute of Anatomy, Ludwig-Maximilians-Universität München, Munich, Germany; ^2^Division of Cell and Developmental Biology, Institute of Biology, Sächsische Inkubator für Klinische Translation (SIKT), University of Leipzig, Leipzig, Germany

**Keywords:** desmosome, keratin, desmoglein, atomic force microscopy, p38MAPK

## Abstract

Keratins are crucial for the anchorage of desmosomes. Severe alterations of keratin organization and detachment of filaments from the desmosomal plaque occur in the autoimmune dermatoses pemphigus vulgaris and pemphigus foliaceus (PF), which are mainly caused by autoantibodies against desmoglein (Dsg) 1 and 3. Keratin alterations are a structural hallmark in pemphigus pathogenesis and correlate with loss of intercellular adhesion. However, the significance for autoantibody-induced loss of intercellular adhesion is largely unknown. In wild-type (wt) murine keratinocytes, pemphigus autoantibodies induced keratin filament retraction. Under the same conditions, we used murine keratinocytes lacking all keratin filaments (KtyII k.o.) as a model system to dissect the role of keratins in pemphigus. KtyII k.o. cells show compromised intercellular adhesion without antibody (Ab) treatment, which was not impaired further by pathogenic pemphigus autoantibodies. Nevertheless, direct activation of p38MAPK *via* anisomycin further decreased intercellular adhesion indicating that cell cohesion was not completely abrogated in the absence of keratins. Direct inhibition of Dsg3, but not of Dsg1, interaction *via* pathogenic autoantibodies as revealed by atomic force microscopy was detectable in both cell lines demonstrating that keratins are not required for this phenomenon. However, PF-IgG shifted Dsg1-binding events from cell borders toward the free cell surface in wt cells. This led to a distribution pattern of Dsg1-binding events similar to KtyII k.o. cells under resting conditions. In keratin-deficient keratinocytes, PF-IgG impaired Dsg1-binding strength, which was not different from wt cells under resting conditions. In addition, pathogenic autoantibodies were capable of activating p38MAPK in both KtyII wt and k.o. cells, the latter of which already displayed robust p38MAPK activation under resting conditions. Since inhibition of p38MAPK blocked autoantibody-induced loss of intercellular adhesion in wt cells and restored baseline cell cohesion in keratin-deficient cells, we conclude that p38MAPK signaling is (i) critical for regulation of cell adhesion, (ii) regulated by keratins, and (iii) targets both keratin-dependent and -independent mechanisms.

## Introduction

Desmosomes are highly organized protein complexes required for proper intercellular adhesion especially in tissues which are constantly exposed to mechanical stress, such as the heart and the epidermis. They are composed of desmosomal cadherins which maintain the strong intercellular adhesion with their extracellular domains (EC), thus bridging the intercellular cleft, and plaque proteins connecting the desmosomal cadherins to the intermediate filament cytoskeleton (1–3).

Pemphigus is a life-threatening autoimmune dermatosis in which autoantibodies directed against the desmosomal cadherins desmoglein (Dsg) 1 and 3 lead to a flaccid blistering of the skin and mucous membranes (4, 5). On a morphological level, blistering occurs by separation of epidermal layers either suprabasal in pemphigus vulgaris (PV) or superficially in pemphigus foliaceus (PF) which represent the two main clinical manifestations of the disease (6). In PF, blisters are restricted to the skin and only Dsg1 autoantibodies occur. By contrast, in PV erosions additionally affect mucous membranes especially of the oral cavity. Blisters in PV are primarily caused by autoantibodies against both, Dsg1 and 3 (7). Thus, autoantibody profiles largely correlate with the clinical phenotype, a phenomenon which was proposed to be explained at least in part by autoantibody-specific cellular signaling patterns (8). In addition to signaling pathways which apparently are crucial for pemphigus pathogenesis (7, 9) direct inhibition of Dsg interactions by autoantibodies was described for Dsg3 but not for Dsg1 (10–12). Furthermore, the typical morphological hallmark of keratin filament retraction from cell borders is a common feature of all clinical phenotypes and can be detected in pemphigus models *in vitro* (13–16) as well as *ex vivo* and in patients’ lesions (17–21). Keratins, the constituents of intermediate filaments in the epidermis, are crucial for proper desmosomal adhesion and retraction of the keratin cytoskeleton correlated with loss of intercellular adhesion induced by pemphigus autoantibodies (11, 22, 23). They, furthermore, account for the mechanical properties of keratinocytes (24) and are involved in the regulation of important signaling pathways for desmosomal adhesion, such as protein kinase C (PKC) and p38 mitogen-activated protein kinase (p38MAPK) both of which also regulate Dsg3-binding properties in a keratin-dependent fashion (22, 23, 25).

In these settings, the exact mechanism and contribution of alterations of the keratin cytoskeleton to loss of intercellular adhesion in pemphigus is not well characterized. Thus, we here use murine keratinocytes lacking all keratins to dissect the contribution of keratins in pemphigus pathogenesis. With this approach we demonstrate that keratins differentially regulate the binding properties of the two major antigens for autoantibodies in pemphigus, Dsg1 and 3. Moreover, we observed that p38MAPK underlies a keratin-mediated regulation, which is crucial for loss of intercellular adhesion in pemphigus.

## Materials and Methods

### Cell Culture and Reagents

In this study, murine keratinocytes (KtyII) isolated from wild-type (KtyII wt) and keratin cluster II knockout (KtyII k.o.) were used. Cells were immortalized as described elsewhere in detail (22). Cells were grown in complete FAD media (0.05 mM CaCl_2_) on collagen I-coated culture dishes (rat tail; BD). For all experiments, cells were grown to confluency before switching them to high Ca^2+^ (1.2 mM) for 48 h to induce proper differentiation and usage for experiments. For fluorescence recovery after photobleaching (FRAP) experiments, cells were transient transfected at 70% confluency with pEGFP-C1-*Dsg3* (kindly provided by Dr. Yasushi Hanakawa, Ehime University School of Medicine, Japan) using Lipofectamine 3000 (Invitrogen, Carlsbad, CA, USA) according to manufacturers’ protocol. 24 h after transfection, cells were switched to high Ca^2+^ (1.2 mM) and grown for further 48 h before the experiments. Activity of p38MAPK was modulated using either p38 inhibitors SB202190 (Merck, Darmstadt, Germany) and SB203580 (Sigma Aldrich, Munich, Germany) (both 30 µM) or p38 activator anisomycin (60 µM) (Sigma Aldrich, Munich, Germany).

### Purification of Recombinant Dsg Fc Constructs

Dsg1- and Dsg3-Fc constructs containing the full extracellular domain of the respective Dsg were stably expressed in Chinese hamster ovary cells (CHO-cells). Purification was performed as described elsewhere in detail (10). Briefly, transfected CHO-cells were grown to confluence, supernatants were collected and recombinant proteins were isolated using Protein A Agarose (Life Technologies). To test purity and specificity Coomassie staining and Western blotting using anti Dsg1-monoclonal antibody (mAb) (p124, Progen, Heidelberg, Germany) and anti Dsg3-mAb (clone5G11; Life Technologies) which both detect the extracellular domain of the respective Dsg were conducted (data not shown).

### Purification of Patients IgG Fractions and Antibodies (Abs)

Serum of PV patients was provided by Enno Schmidt (Department of Dermatology, University of Lübeck). Sera were used with informed and written consent and under approval of the local ethic committee (number: AZ12-178). All patients had an active disease at the time of collection including lesions of the skin and the mucous membranes. ELISA scores are given in Table 1.

**Table 1:** ELISA score of pemphigus vulgaris (PV)-IgG fraction.

	Dsg1	Dsg3
PV1-IgG	1,207	3,906
PV2-IgG	212.27	181.440
Pemphigus foliaceus (PF)-IgG	215.34	8.2^a^

*^a^Beneath relevant threshold*.

Purification of IgG fraction from pemphigus patients (PV-IgG) or healthy volunteer (control-IgG) was conducted as described elsewhere (10, 12, 26) using Protein A Agarose (Life Technologies). The pathogenic monoclonal Dsg3 Ab, AK23 (Biozol, Eching, Germany) was used at a concentration of 75 µg/ml.

### Immunostaining

Murine keratinocytes were used 48 h after Ca^2+^ switch and 72 h after transfection for the FRAP and respective immunofluorescence experiments. Cells were fixed with freshly prepared 4% paraformaldehyde for 20 min, permeabilized with 1% Triton X-100 for 10 min and blocked with 10% normal goat serum/1% bovine serum albumin. Cytokeratin 14 mAb (LL002, Abcam, Cambridge, UK) was used as primary Ab. As secondary Ab Cy3-labeled goat anti-mouse Ab was used (Dianova, Hamburg, Germany). Furthermore, Alexa 488-phalloidin (Invitrogen, Carlsbad, CA, USA) and DAPI (Roche, Mannheim, Germany) were used to visualize the actin cytoskeleton and the nuclei respectively. Images were recorded using a Leica SP5 confocal microscopy with a 63× NA 1.4 PL APO objective controlled by LAS AF software (Leica, Mannheim, Germany). For some experiments, *z*-stacks were recorded, and pictures represent maximum intensity projections. For quantification of keratin retraction keratin 14 fluorescence intensity was measured at small areas in close proximity to the cell border and above the nucleus, and a ratio was calculated to quantify keratin filament retraction (Figure S1A in Supplementary Material).

### Dispase-Based Keratinocytes Dissociation Assay

For dissociation assay, cells were grown in high Ca^2+^ medium for 48 h after confluence. Dispase assay was conducted as described in detail before (25, 27). Briefly, cells exposed to different conditions were removed from well bottom using a mixture of Dispase II (Sigma Aldrich) and 1% collagenase I (Thermo Fisher Scientific). After application of defined shear stress by pipetting the monolayers with a 1 ml pipette the resulting fragments were counted. The latter represent an inverse measure for intercellular adhesion.

### Biotinylation Assay and Western Blotting

Cell surface biotinylation was conducted as described before (11, 25). In brief, cell monolayers were incubated with 0.25 mM of membrane-impermeable EZ-Link Sulfo-NHS-Biotin (Thermo Fisher Scientific, Waltham, MA, USA) on ice and rinsed in ice-cold PBS containing 100 mM glycin. Cells were lysed in PIPES buffer (50 mM NaCl, 10 mM PIPES, 3 mM MgCl_2_, 1% Triton X-100, protease inhibitors) and centrifuged. Supernatants were collected, and pull-down of biotinylated molecules was carried out using NeutrAvidin (HighCapacity)-agarose (Thermo Fisher Scientific). Precipitated molecules were suspended in 3× Laemmli buffer with 50 mM dithiothreitol (AppliChem) and subjected to Western blotting.

For whole cell lysates SDS buffer (25 mmol/l HEPES, 2 mmol EDTA, 25 mmol/l NaF and 1% sodiumdodecylsulfate, and pH 7.4) was used. Western blotting was performed according to standard protocol (27).

### Fluorescence Recovery After Photobleaching

For all FRAP experiments, KtyII cells were seeded in 8-well imaging chambers (Ibidi, Martinsried, Germany). Cells were transfected with pEGFP-C1-Dsg3 as described in cell culture section. FRAP experiments were conducted with the FRAP wizard software on a Leica SP5 confocal microscopy with a 63× NA 1.4 PL APO objective at 37°C as described before (25, 28). The Dsg3-GFP signal was bleached at cell border areas of two adjacent cells with the 488 nm line of an Argon laser at 100% transmission. Fluorescence recovery was monitored during the following 3 min and fluorescence intensities were analyzed. The immobile fraction was determined using the FRAP wizard.

### Atomic Force Microscopy (AFM) Measurements

A NanoWizard^®^ 3 AFM (JPK Instruments, Berlin, Germany) mounted on an inverted optical microscope (Carl Zeiss, Jena, Germany) was used throughout all experiments. The setup was described in detail before and allows the selection of the scanning areas by usage of an optical image acquired with a 63× objective (11, 25, 29). All measurements were accomplished at 37°C in cell culture medium containing 1.2 mM Ca^2+^.

For all experiments pyramidal-shaped D-Tips of Si_3_N_4_ MLCT cantilevers (Bruker, Mannheim, Germany) with a nominal spring constant of 0.03 N/m and tip radius of 20 nm were functionalized with Dsg1- or 3-Fc constructs as described before (30). Briefly, a flexible heterobifunctional acetal-polyethylenglycol (synthetized by the Hermann Gruber Lab, Institute of Biophysics, Linz, Austria) was interspaced between the tip and the purified Fc construct whose concentration was adjusted to 0.15 mg/ml. For measurements on living murine keratinocytes a protocol which was recently developed in our group (29) was used. AFM was run in quantitative imaging (QI) mode for overview images or force mapping (FM) mode for measurement and characterization of Dsg binding properties. For the latter mode, a force map consisted of 1,200 pixels with each pixel representing one force–distance cycle that covered an area of 6 μm × 2 μm along cell borders or 4 μm × 2 μm above the nucleus. Settings of the respective modes can be found in Table 2.

**Table 2:** Atomic force microscopy settings.

Settings	Quantitative imaging mode	Force mapping mode
Setpoint	0.5 nN	0.5 nN
*Z*-length	1.5 µm	1.5 µm
Pulling speed	50 µm/s	10 µm/s
Resting contact time	–	0.1 s

Resulting force–distance curves were analyzed with regard to topography and adhesive properties of specific surface molecules (31).

For Ab experiments, the respective Abs were incubated solely on the cell to avoid binding to the scanning tip. After the incubation period, cells were extensively washed to remove unbound Abs and reprobed by AFM. For characterization of molecule distribution at the cell border areas a distribution coefficient was calculated as described before (32). Briefly, a ratio of number of binding events per area along the elevated cell borders (Figure S1B in Supplementary Material, green area) and in the surrounding cell surface (Figure S1B in Supplementary Material, red area) was calculated, in which values higher than 1 demonstrate increased localization of molecules along the cell borders.

### Data Analysis and Statistics

For image processing, Adobe Photoshop CS5 (Adobe, Dublin, Ireland) was used. AFM images and data analysis of force–distance curves were done on JPK Data Processing Software (JPK Instruments). For further calculation of the analyzed AFM data with regard to unbinding forces, peak fitting, and step position Origin Pro 2016, 93G (Northampton, MA, USA) was used. In addition, Origin was utilized for comparison of data values with a paired Student’s *t*-test (two sample groups) or one-way analysis of variance following Bonferroni correction (more than two groups), respectively. Error bars given in the figures are mean ± SD for all diagrams depicting unbinding forces ± SEM for all other experiments. Significance was presumed at a *p*-value < 0.05.

## Results

### Keratin Deficiency Reduces Effect of Pathogenic Autoantibodies on Intercellular Adhesion

**Figure 1:**
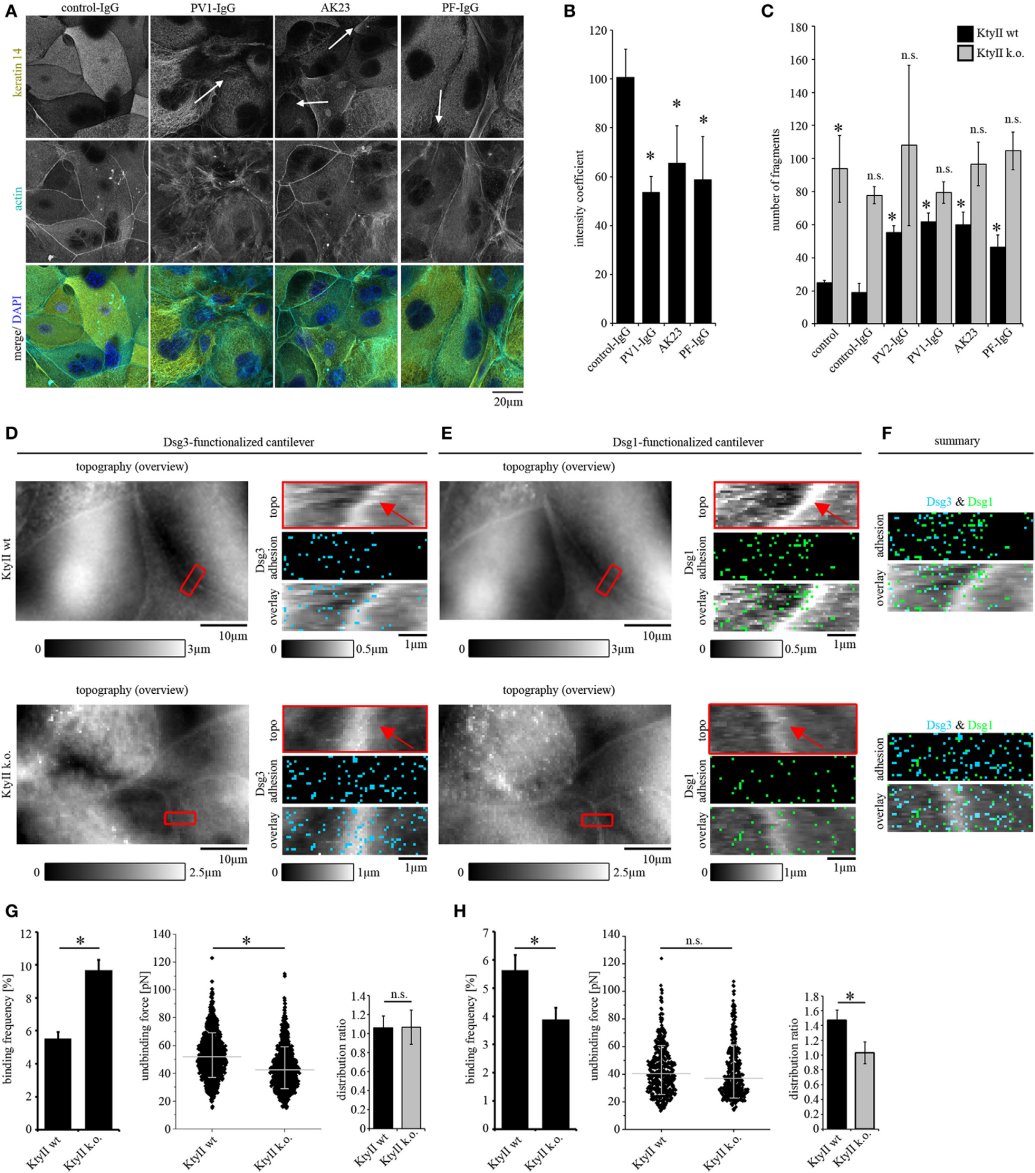
Keratin retraction in murine keratinocytes and regulation of desmoglein binding properties through keratins. **(A)** Immunostaining of wild-type murine keratinocytes (KtyII wt) using keratin 14 monoclonal antibody and Alexa488-phalloidin for actin staining. Control-IgG treated keratinocytes show a dense keratin network throughout the whole cell whereas keratin filament retraction (arrows) and formation of thick filament bundles were detectable after treatment with pemphigus vulgaris (PV)-IgG, pemphigus foliaceus (PF)-IgG, and AK23. Pictures are representatives of *n* > 4. **(B)** Quantification of keratin retraction using an intensity coefficient confirms occurrence of keratin retraction in all autoantibody treated conditions. **(C)** Dissociation assay of KtyII wt and k.o. keratinocytes reveals that keratin-deficient keratinocytes show impaired intercellular adhesion. Treatment with pathogenic autoantibodies (PF-IgG, PV1- and PV2-IgG, and AK23) reduced intercellular adhesion in wild-type (wt) but not in KtyII k.o. cells. *n* = 5, *p > 0.05 vs. wt control and wt control-IgG. **(D,E)** Atomic force microscopy adhesion measurements using Dsg3-Fc- or Dsg1-Fc-functionalized cantilevers on the same scanning area. In adhesion panels, each blue or green pixel represents a specific Dsg3- or Dsg1-binding event, respectively. **(F)** Merged panels of adhesion measurements reveal distinct clustering of Dsg1- and 3-binding events. **(G,H)** Analysis of binding frequency and unbinding forces of Dsg1 and 3 interactions. *n* = 6 from ≥3 independent coating procedures, 1,200 force–distance curves/adhesion maps (*p < 0.05).

Keratin alterations are morphological hallmarks of pemphigus and correlate with loss of intercellular adhesion in human keratinocytes (11). Wild-type murine keratinocytes (KtyII wt) were used to test whether pathogenic autoantibodies are able to induce comparable changes of the keratin cytoskeleton in murine cells. Keratinocytes were treated either with control-IgG of healthy volunteers or with pathogenic autoantibodies for 24 h and subjected to immunostaining for keratin14 or keratinocyte dissociation assay, respectively (Figures 1A,B). We used AK23, a pathogenic monoclonal Ab derived from a PV mouse model, which is specific for Dsg3 (33), PF-IgG containing Ab against Dsg1 as well as PV-IgG with Abs against Dsg1 and 3 (Table 1). In control-IgG treated cells, the keratin cytoskeleton formed a dense network throughout the cells which is composed of delicate fibers and covers the cell periphery (Figure 1A). Actin was labeled to delineate the cell periphery and DAPI was included to stain nuclei (Figure 1A). By contrast, after treatment with all pathogenic autoantibodies keratin filament bundles were thicker and more irregular throughout and were retracted from some segments of cell borders (Figure 1A, arrows). Keratin retraction was quantified as described in materials and methods. In control-IgG treated cells, the coefficient was around 1 indicating a homogeneous distribution of the keratin network throughout the whole cell. The coefficient was significantly reduced after treatment with all pathogenic autoantibodies indicating a reduced fluorescent signal at cell border areas and thus confirms the occurrence of keratin retraction (Figure 1B).

To further dissect the role of keratins for loss of intercellular adhesion in pemphigus, KtyII k.o. cells were compared with wt monolayers in dissociation assays (Figure S1C in Supplementary Material). Under control conditions and after incubation with control-IgG, KtyII k.o. cells showed a significantly impaired intercellular adhesion compared with wt similar as shown before (22, 23, 25) (Figure 1C). Interestingly, treatment with AK23, PV-IgG or PF-IgG did not further compromise intercellular adhesion in KtyII k.o. cells whereas a significant reduction in wt cells was observed (Figure 1C). Given that anisomycin-mediated activation of p38MAPK as shown below (Figure 4B) was efficient to strongly reduce adhesion in keratin-deficient cells, these data indicate that keratins are important for the loss of intercellular adhesion in pemphigus.

### Keratins Differentially Regulate Dsg-Binding Properties

Keratin filament alterations in response to autoantibodies were accompanied by depletion of Dsg3 from the cell membrane (8, 11). In a recent study, it was shown that keratins regulate Dsg3-binding properties through signaling (25). Thus, we next studied distribution, binding frequency, and binding strength of Dsg1 and Dsg3 by AFM in parallel. Dsgs can interact homo- and heterophilic (34–36). However, in our last studies by comparing parallel experiments under cell-free conditions and on living keratinocytes as well as by using isoform-specific inhibitory Abs, we predominantly detected homophilic interactions (25, 29, 32). A modified cantilever holder setup allowed the measurement at the same area with scanning tips functionalized with Dsg1 or Dsg3, respectively. First, topography overview images spanning an area of 50 μm × 30 μm were performed using QI mode (Figures 1D,E). In both cell lines, cell surfaces exhibited a reticular structure, and cell borders could be identified clearly by an elevated region (25) (Figures 1D,E, red arrows). Defined areas along cell borders were chosen for adhesion measurements (Figures 1D,E, red rectangles) and probed using FM mode with a pulling speed of 10 µm/s and a resting contact time of 0,1 s. Same areas along the cell border were chosen with both cantilevers after respective overview imaging to compare Dsg1 and 3 localization. In adhesion panels, each blue and green pixel represents a specific binding event of Dsg3 and Dsg1, respectively (Figures 1D–F). Evaluation of binding frequencies revealed a higher binding frequency for Dsg3 in keratin-deficient keratinocytes (Figures 1D,G) whereas the Dsg1-binding frequency was significantly reduced (Figures 1E,H). By contrast, binding forces of Dsg3 were reduced in KtyII k.o. cells as shown previously (25) (Figures 1D,G) whereas no difference in binding strength was observed for Dsg1 (Figures 1E,H). Finally, we analyzed the distribution of binding events using a distribution coefficient (see Material and Methods; Figure S1B in Supplementary Material) in which values >1 indicated higher binding frequency along the cell border. In accordance with former studies, Dsg3 shows a uniform distribution in both cell lines (25, 29) (Figures 1D,F,G). However, Dsg1-binding events were localized along cell borders (Figures 1E,F,H) in wt cells, whereas distribution was uniform in KtyII k.o. cells (Figures 1E,F,H) suggesting that keratins are important for proper localization of Dsg1 at cell junctions. Moreover, keratins differentially modulate Dsg binding properties.

### Direct Inhibition of Dsg3 Single Molecule Interactions Occurs in Keratin-Deficient Keratinocytes

**Figure 2:**
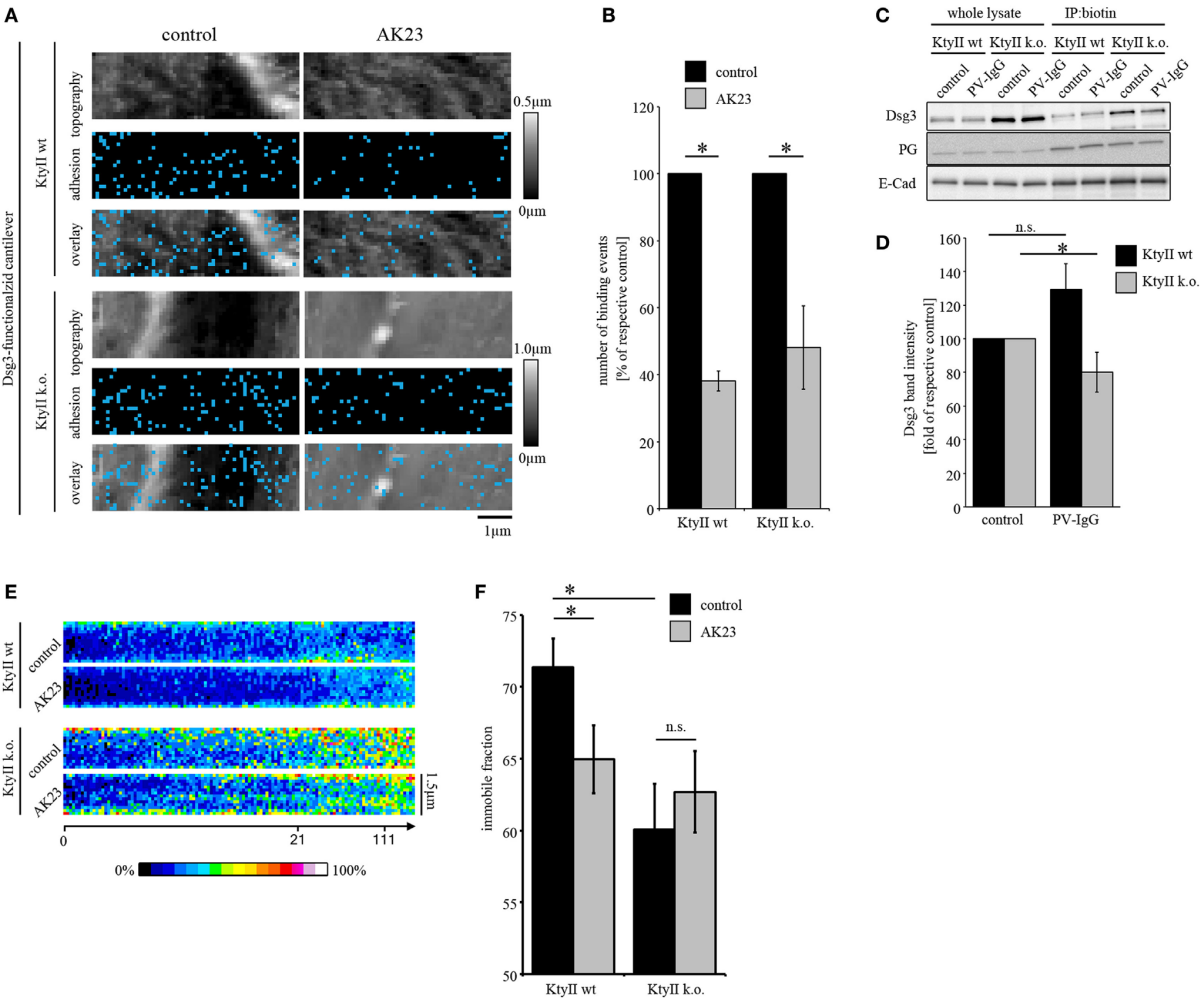
Keratin-deficiency accelerates depletion of Dsg3. **(A,B)** Dsg3 adhesion measurements on KtyII wt and k.o. cells using AK23. Treatment of KtyII wt and k.o. cells with AK23 for 1 h reduced Dsg3 binding to comparable extent. *n* = 6 from ≥3 independent coating procedures, 1,200 force–distance curves/adhesion maps (*p < 0.05). **(C,D)** Cell surface biotinylation delineates depletion of Dsg3 in keratin-deficient keratinocytes after 1 h of pemphigus vulgaris (PV) 1-IgG treatment but not in wild-type keratinocytes. Representative of *n* = 5, *p < 0.05 vs. respective control. **(E)** Kymographs of fluorescence recovery after photobleaching experiments using Dsg3-eGFP-transfected KtyII wt and k.o. keratinocytes. **(F)** Immobile fractions of Dsg3-eGFP in KtyII wt and k.o. cells under basal conditions and after incubation with AK23 incubation for 1 h; *n* = 7, 5 cell borders/experiment; *p < 0.05.

Direct inhibition of Dsg3 interactions is a well-characterized phenomenon in pemphigus which occurs fast after binding of pathogenic autoantibodies but alone is not sufficient to cause complete loss of cell cohesion (5, 11, 26). To investigate the role of keratins for direct inhibition, we treated both cell lines with AK23 which is directed against the extracellular domain (EC) 1 of Dsg3 and was reported to interfere with Dsg3 interaction under cell-free conditions as well as in living keratinocytes (10, 11, 36). To avoid Ab binding to the AFM cantilever, AK23 was incubated on cells for 1 h in absence of cantilevers, washed extensively after incubation with fresh media to remove unbound Abs, and cells were reprobed. Doing so, small areas along the cell borders were chosen and measured before and after AK23 incubation. AK23 reduced Dsg3-binding frequency in both cell lines to a comparable extent suggesting that loss of keratins do not effect autoantibody-induced direct inhibition of Dsg3 interaction (Figures 2A,B). In line with this, distribution of the remaining binding events was not changed (Figures 2A,B).

### Keratin Deficiency Accelerates Depletion of Dsg3

Next, we investigated depletion of Dsg3 which accompanied loss of intercellular adhesion and was linked to several signaling pathways such as p38MAPK in models of pemphigus (19, 37). As reported previously, keratin-deficient keratinocytes reveal a higher expression level of Dsg3 (25) (Figure 2C). Cells were treated with PV-IgG for 1 h and subjected to a surface biotinylation assay. In whole cell lysates, no reduction of Dsg3 levels was detectable in both cell lines (Figure 2C). However, in the surface membrane pool harvested *via* immunoprecipitation of biotin PV-IgG induced a significant depletion of Dsg3 in KtyII k.o. cells but not wt monolayers after 1 h of incubation (Figures 2C,D) indicating that the turnover of Dsgs is altered in keratin-deficient keratinocytes. This may be explained by enhanced mobility of Dsg3 when keratins are missing (25). Thus, we performed FRAP experiments on Dsg3-pEGFP-transfected KtyII cells using AK23. Indeed, KtyII k.o. cells revealed a reduced immobile fraction indicating higher mobility of Dsg3 molecules under basal conditions (Figures 2E,F). However, incubation of AK23 enhanced Dsg3 mobility in wt cells only suggesting that higher mobility resulted from keratin uncoupling (Figures 2E,F).

### PF-IgG and PV-IgG Cause Redistribution of Dsg1-Binding Events and Subsequently Reduce Dsg1-Binding Strength

Under cell-free conditions, direct inhibition of Dsg interaction was observed for Dsg3 but not for Dsg1 (8, 10, 12). To test whether direct inhibition of Dsg1 occurs on living keratinocytes, we performed AFM adhesion measurements using PF-IgG. Small areas along cell borders and on the cell surface above the nucleus (Figure S2A in Supplementary Material) were chosen and probed in FM mode.

**Figure 3:**
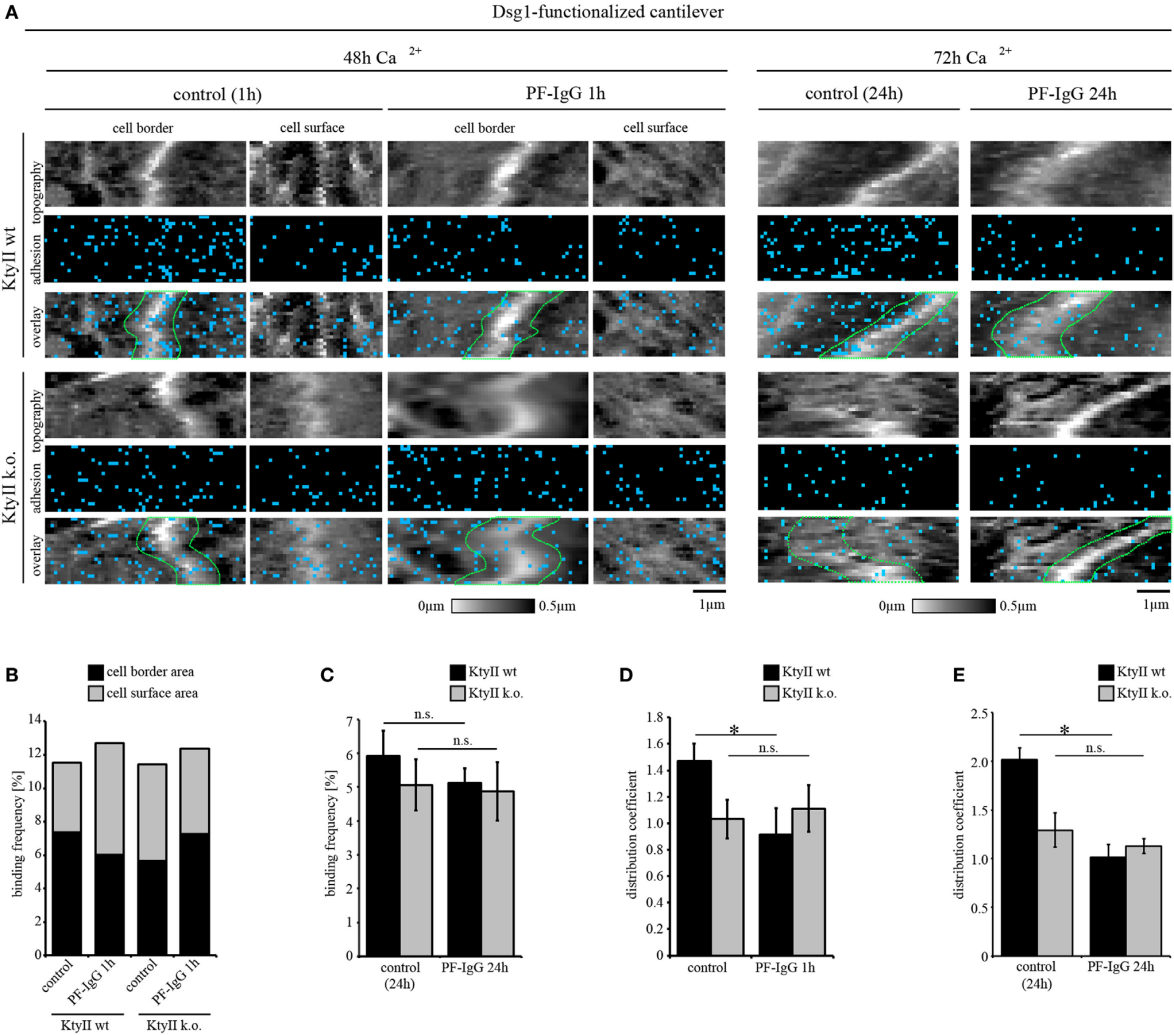

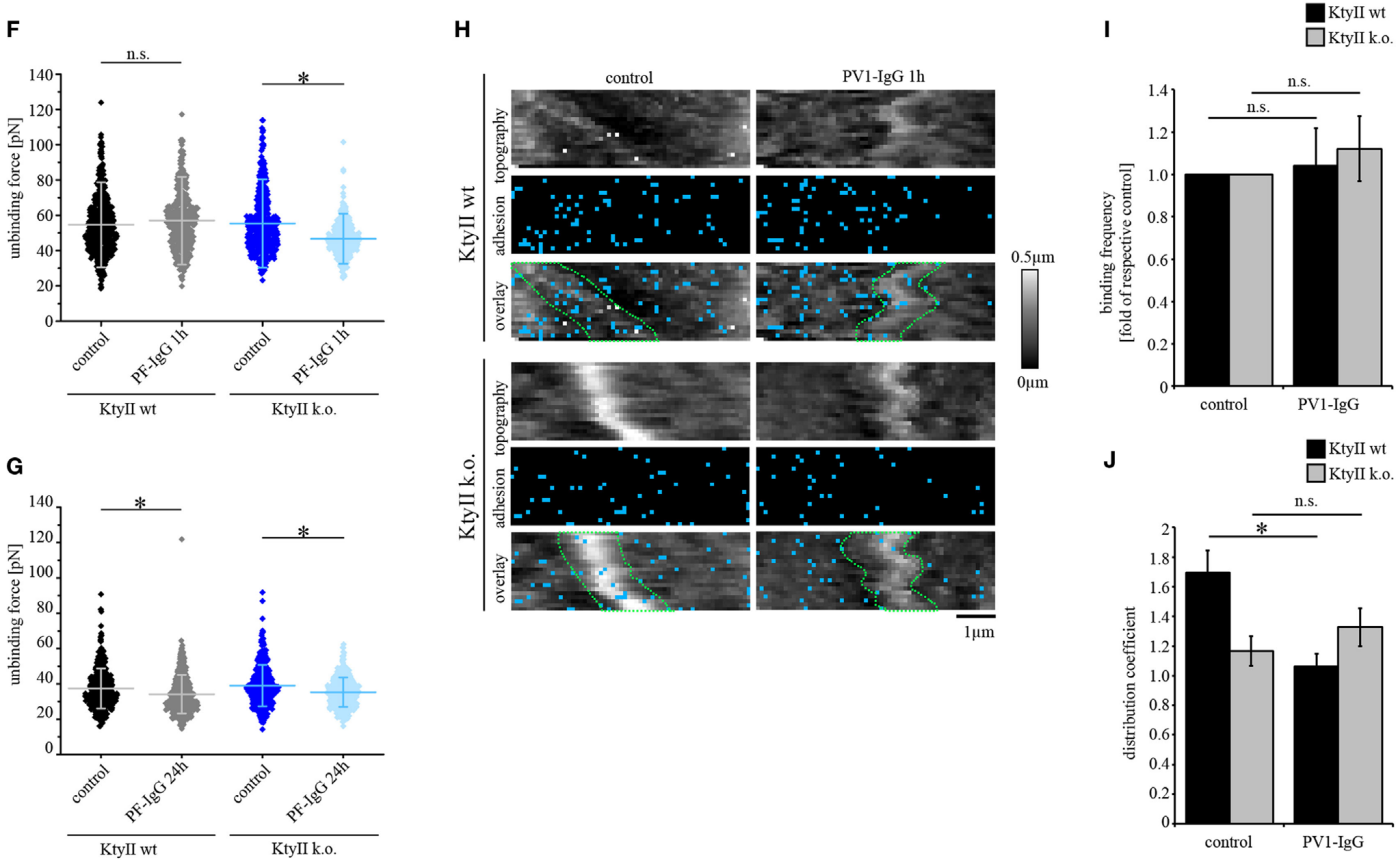
aDsg1 antibodies (Abs) causes a keratin-dependent redistribution of Dsg1. **(A)** Atomic force microscopy (AFM) adhesion measurements using Dsg1-Fc-functionalized tips at small areas along cell borders and on the cell surface above the nucleus. Pemphigus foliaceus (PF)-IgG for 1 h and 24 h induced redistribution of Dsg1 from cell junctions in wild type. **(B)** Dsg1-binding frequencies under control condition and after 1 h of PF-IgG treatment show no direct inhibition of binding events. **(C)** 24 h of PF-IgG treatment slightly reduced Dsg1-binding frequency in KtyII wt and k.o. cells. **(D,E)** Distribution coefficient of Dsg1-binding events under control conditions and after 1 and 24 h of PF-IgG incubation. *n* = 4 from 3 independent coating procedures, 1,200 force–distance curves/adhesion map (*p < 0.05). **(F,G)** Unbinding forces of Dsg1-binding events under control conditions and after 1 or 24 h of PF-IgG incubation. *n* = 4 from 3 independent coating procedures, 1,200 force–distance curves/adhesion map (*p < 0.05). **(H)** AFM adhesion measurements using Dsg1-Fc functionalized tips in KtyII wt and k.o. cells under control conditions or after incubation with pemphigus vulgaris (PV) 1-IgG containing aDsg1 Abs for 1 h. **(I)** Dsg1-binding frequency did not change after 1 h of PV-IgG treatment in both cell lines. **(J)** Distribution of Dsg1-binding events was altered in KtyII wt but not in KtyII k.o. cells after PV1-IgG treatment. *n* = 4 from 3 independent coating procedures, 1,200 force–distance curves/adhesion map (*p < 0.05).

As outlined earlier, under control conditions Dsg1-binding events exhibited clusters along the cell borders in wt cells and uniform distribution in KtyII k.o. cells. In line with this, less Dsg1-binding events were detected on the cell surface of wt cells compared with cell borders whereas no difference in binding frequency was observed in KtyII k.o. (Figures 3A,B; Figure S2B in Supplementary Material). Interestingly, the sum of binding frequencies along cell borders and on the cell surface was similar in both lines (Figures 3A,B), indicating that attachment to desmosomes is crucial for the localization of Dsg1 at cell borders. To test the specificity of Dsg1-binding events, we used a monoclonal aDsg1, which was capable of blocking homophilic interactions under cell-free conditions (25, 36). Incubation of the Ab for 1 h on the cells in the absence of cantilevers revealed a significant reduction of binding frequency in both cell lines indicating that we measured specific Dsg1 interactions (Figures S2C,D in Supplementary Material).

Next, we tested whether PF-IgG can induce direct inhibition of Dsg1 binding. Interestingly, PF-IgG did not reduce Dsg1-binding frequency in both cell lines but led to a redistribution of Dsg1-binding events from cell borders in wt cells (Figure 3A, cell borders surrounded by dashed green lines, Figures 3B,D) as reflected by the distribution coefficient (Figure 3D). However, no shift was observed in keratin-deficient keratinocytes where Dsg1 was less localized at cell borders under resting conditions (Figures 3A,B,D) suggesting that keratin uncoupling may account for this phenomenon. By contrast, after PF-IgG treatment for 1 h Dsg1-binding strength was reduced in KtyII k.o. cells only (Figure 3F). Thus, we assumed a cascade in which redistribution of Dsg1-binding events occurs first, followed by a reduction of Dsg1-binding strength, both of which may contribute to loss of intercellular adhesion. To test this, we incubated wt and k.o. cells with PF-IgG for 24 h. Importantly, 72 h after Ca^2+^-induction, we observed binding properties of Dsg1 similar to that after 48 h (Figure 3A). Incubation with PF-IgG for 24 h did not significantly alter Dsg1-binding frequency (Figure 3C). In agreement with the alterations described above, less Dsg1-binding events localized to cell borders after 24 h of PF-IgG treatment in wt cells (Figure 3E). By contrast, binding forces were reduced in both cell lines after 24 h of PF-IgG treatment (Figure 3G) indicating that relocalization of Dsg1 preceded alterations in Dsg1 adhesive strength.

We confirmed these results for aDsg1 Abs with PV-IgG in Dsg1 adhesion measurements. Similar to PF-IgG, incubation with PV-IgG for 1 h led to no reduction in binding frequency in both lines whereas a redistribution of Dsg1-binding events was present in wt cells only (Figures 3H–J). Thus, the distribution coefficient of Dsg1-binding events was comparable in wt and k.o. cells after treatment with both PF- and PV-IgG (Figures 3D,E,J).

### p38MAPK Activation Induces Keratin Filament Retraction and Leads to Redistribution of Dsg1- and Dsg3-Binding Events

**Figure 4:**
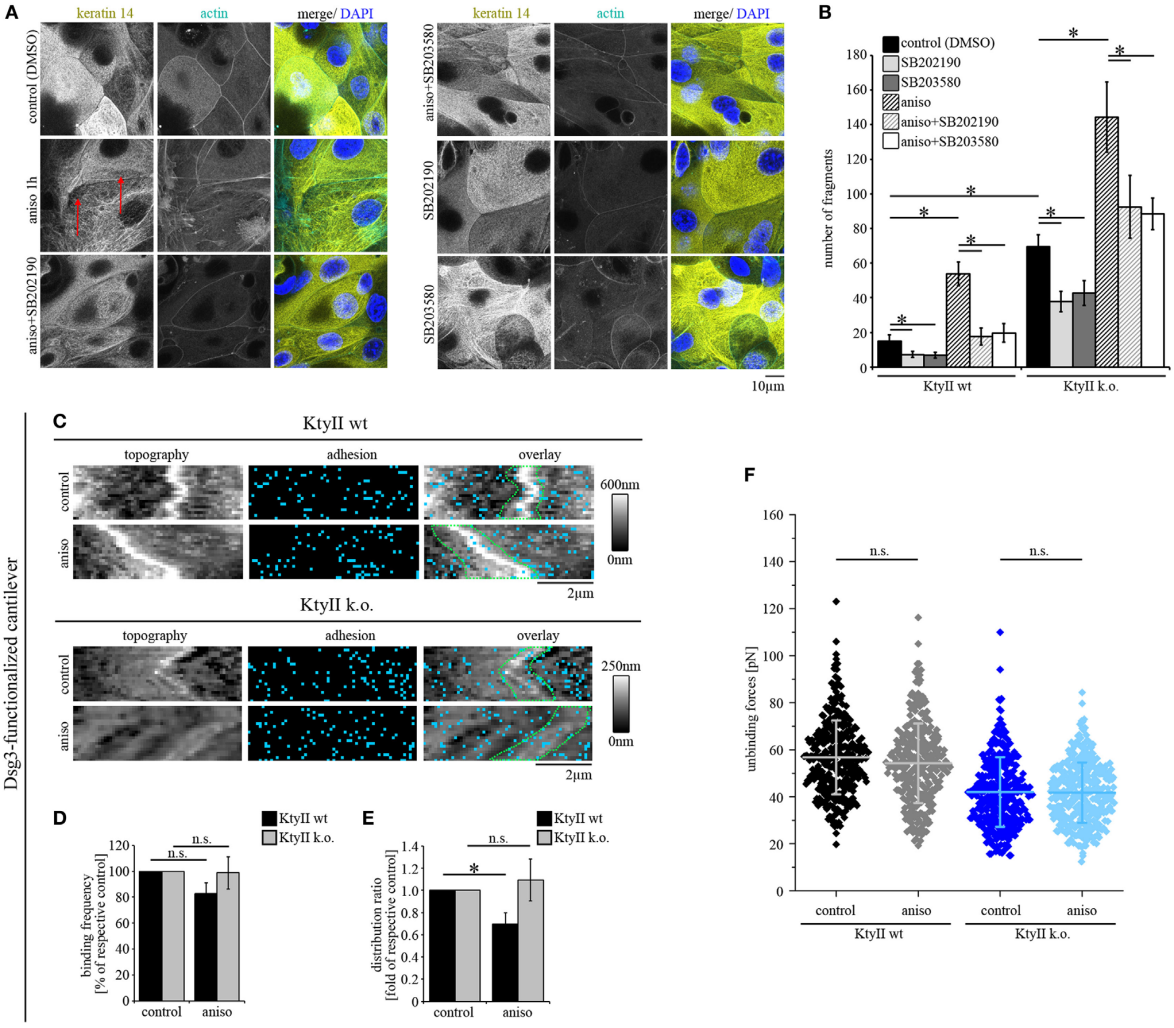

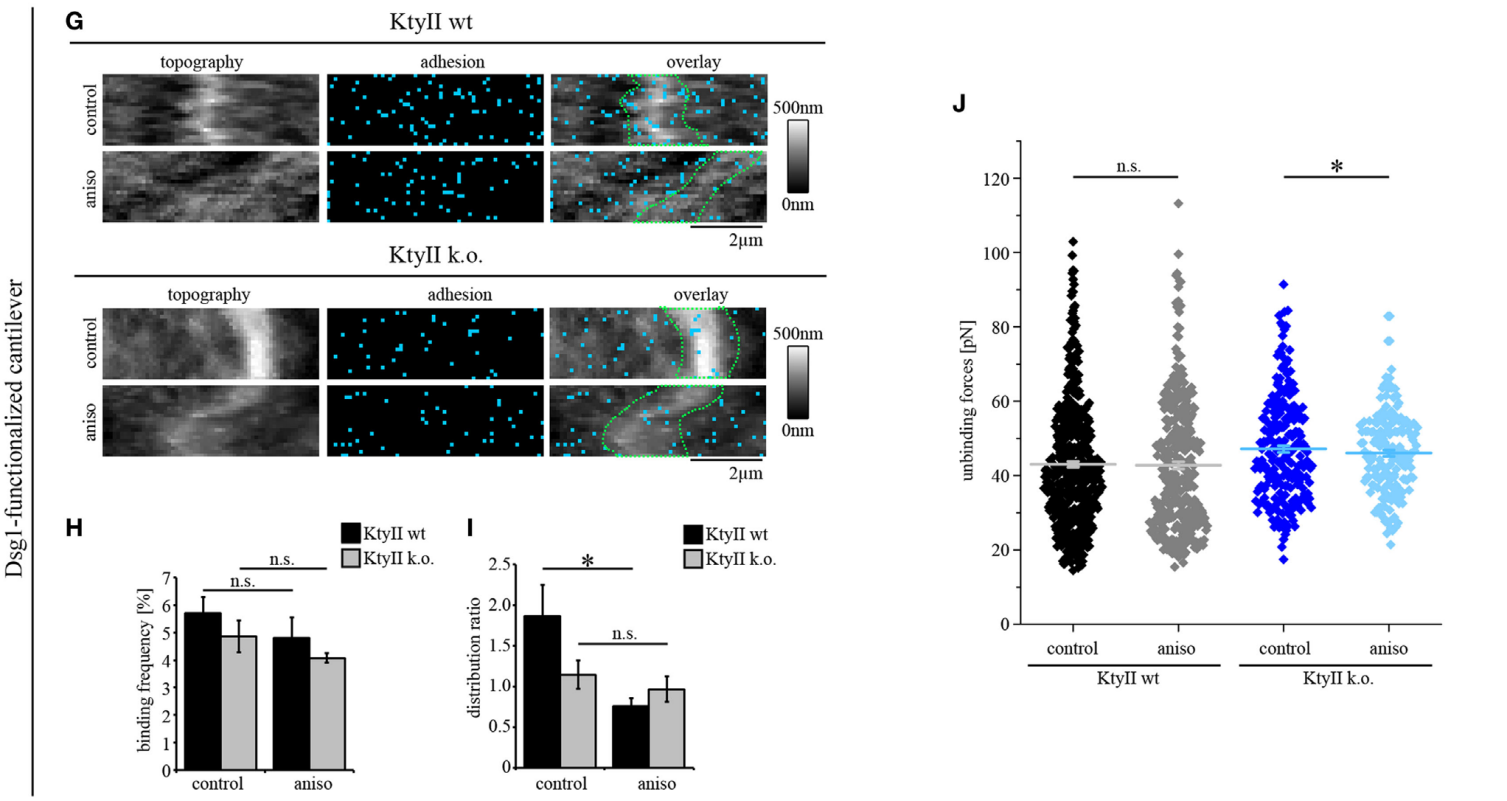
p38MAPK activation induces redistribution of Dsg1- and Dsg3-binding events. **(A)** Immunostaining of keratin 14 and Alexa488-phalloidin staining of actin in KtyII wt after anisomycin treatment for 1 h revealed keratin filament retraction (red arrows), which was blocked by p38MAPK inhibitors SB202190 and SB203580 when preincubated for 1 h. Representative images from *n* = 4. **(B)** Dissociation assay after treatment with anisomycin alone or after preincubation with SB202190 or SB203580 in keratin-deficient keratinocytes compared with wild-type (wt) (*n* > 4, *p < 0.05). **(C–E)** Dsg3 adhesion measurements show no change in binding frequency after anisomycin treatment but reveal a redistribution of binding events away from cell borders in KtyII wt but not in KtyII k.o. cells. **(F)** Unbinding forces of Dsg3 adhesion measurements were not significantly altered in both cell lines. *n* = 4 from 3 independent coating procedures, 1,200 force–distance curves/adhesion map (*p < 0.05). **(G–I)** Dsg1 adhesion measurements revealed redistribution of binding events away from cell borders in KtyII wt but not in KtyII k.o. cells. **(J)** Unbinding forces of Dsg1 adhesion measurements were not significantly altered in wt but reduced in KtyII k.o. cells. *n* = 4 from 3 independent coating procedures, 1,200 force–distance curves/adhesion map (*p < 0.05).

Activation of p38MAPK is a central signaling mechanism induced by pemphigus autoantibody binding and correlates with both, loss of intercellular adhesion and keratin filament retraction (13, 38, 39). Furthermore, inhibition of p38MAPK is capable of restoring intercellular adhesion and keratin filament alterations even under conditions in which direct inhibition of Dsg3 binding is present (11, 38). Furthermore, p38MAPK inhibition prevented Dsg3-binding force reduction in keratin-deficient keratinocytes which show an activation of p38MAPK already under basal conditions (25). Thus, we aimed to investigate how activation of p38MAPK affects Dsg binding properties. Since autoantibodies targeting Dsg3 induce direct inhibition and therefore do not allow characterization of binding properties by autoantibodies (8, 40), we followed a pharmacological approach more specific for p38MAPK and used anisomycin for 1 h to activate p38MAPK similar to previous studies (26). Anisomycin activated p38MAPK after 1 h of incubation both in KtyII k.o. and wt cells which was reduced by the p38MAPK inhibitors SB202190 and SB203580 as revealed by Western blotting (Figure S3A in Supplementary Material). First, we performed immunostaining against keratin 14 to study anisomycin-mediated alterations of keratin filaments in wt cells (Figure 4A). Anisomycin induced keratin filament retraction and reorganization of filaments toward thick bundles similar as described earlier for experiments with pathogenic autoantibodies (Figure 4A, arrows, compare with Figure 1). To confirm that the alterations were induced by anisomycin *via* activation of p38MAPK, we used the p38MAPK inhibitors SB202190 and SB203580, which alone had no effect on the morphology of keratin filaments. Nevertheless, both inhibitors abrogated anisomycin-induced keratin filament alterations (Figure 4A). Next, we tested whether effects of p38MAPK on intercellular adhesion is dependent on keratin filaments using a dissociation assay. Interestingly, anisomycin induced a drastic loss of intercellular adhesion in both KtyII wt and k.o. cells (Figure 4B; Figure S3B in Supplementary Material), which was blocked by co-incubation with p38MAPK inhibitors SB202190 and SB203580. Interestingly, both inhibitors improved basal cell adhesion in wt and keratin-deficient keratinocytes (Figure 4B; Figure S3B in Supplementary Material).

Next, we performed AFM adhesion measurements for Dsg3 and Dsg1 to characterize the effects of p38MAPK activation on Dsg binding properties. Under control conditions Dsg3-binding events were distributed uniformly over the cell surface (Figure 4C). After incubation with anisomycin for 1 h, the overall binding frequency was not changed in both cell lines (Figures 4C,D), but the distribution of Dsg3 was altered in KtyII wt cells and the distribution coefficient indicated less binding events along cell borders (Figures 4C,E). By contrast, distribution was not changed in KtyII k.o. cells (Figures 4C,E). Moreover, aniosomycin did not affect Dsg3 unbinding force (Figure 4F). Next, we performed Dsg1 adhesion measurements. Activation of p38MAPK using anisomycin for 1 h led to a redistribution of Dsg1-binding events from the cell borders only in wt keratinocytes (Figures 4G–I). Furthermore, binding strength of Dsg1 interactions were slightly reduced in KtyII k.o., but not in wt cell after activation of p38MAPK using anisomycin (Figure 4J). Taken together these data indicate that p38MAPK regulates organization of Dsg binding events.

### Effect of p38MAPK on Loss of Intercellular Adhesion Caused by Autoantibodies and Keratin Deficiency

**Figure 5:**
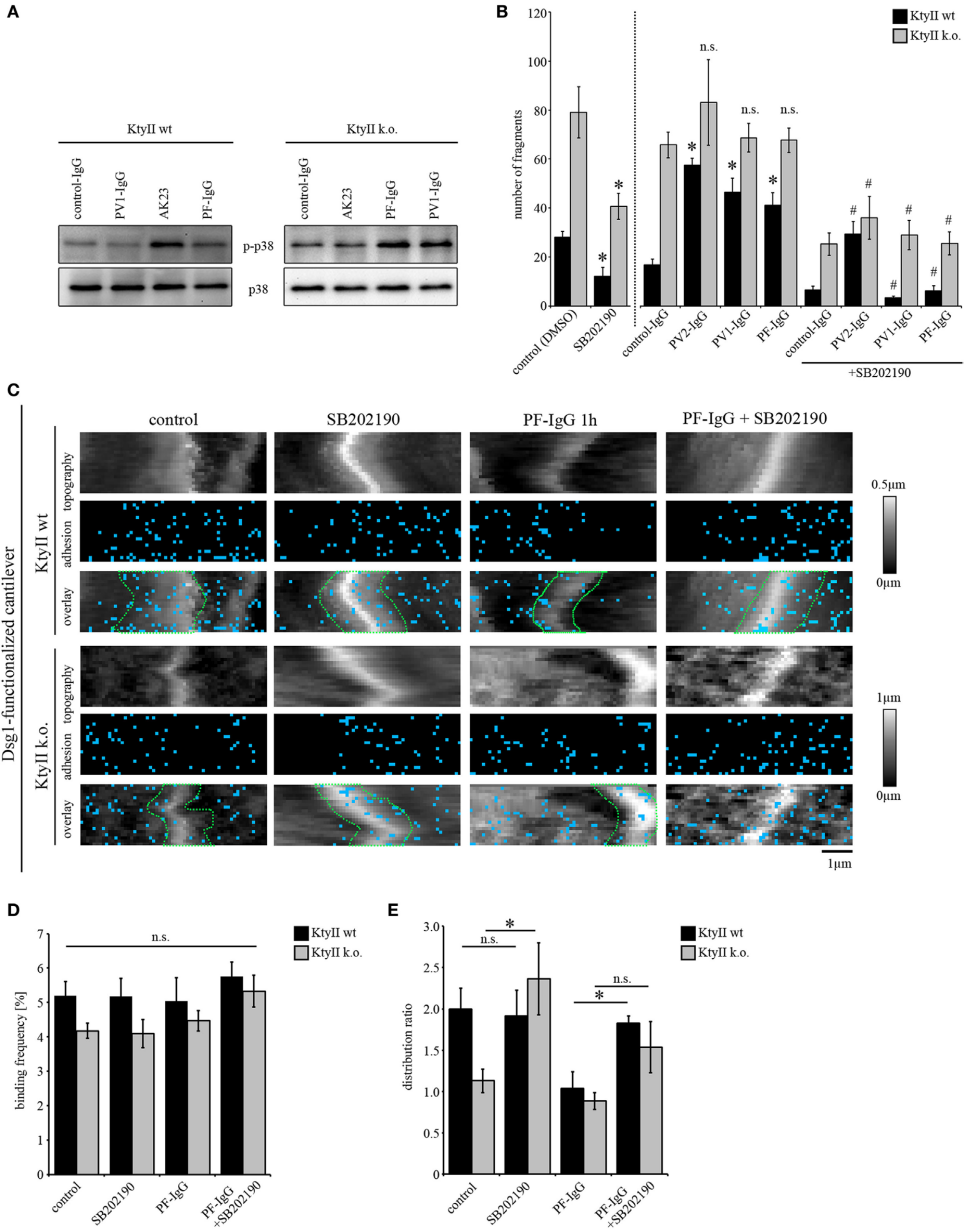
p38MAPK inhibition restores loss of intercellular adhesion caused by autoantibodies and keratin deficiency. **(A)** Western blot showing activation of p38MAPK after treatment with AK23, pemphigus vulgaris (PV) 1-IgG and pemphigus foliaceus (PF)-IgG for 1 h. Representative of *n* > 4. **(B)** Dissociation assay of KtyII wt and k.o. cells after incubation with pathogenic autoantibodies alone or in combination with SB202190 for 24 h (*n* > 5). *p < 0.05 vs. respective control (control panel for SB202190-treated cells and control-IgG for conditions treated with pathogenic autoantibodies); ^#^*p* < 0.05 vs. respective condition treated with pathogenic autoantibodies alone. **(C–E)** Dsg1 adhesion measurements reveal a redistribution of binding events away from cell borders in KtyII wt but not in KtyII k.o. cells after 1 h PF-IgG. SB202190 preincubation for 1 h blocked PF-IgG-induced redistribution in KtyII wt cells and alone caused redistribution of Dsg1-binding events to cell borders in k.o. cells. Binding frequencies were not altered in all conditions. *n* = 3 from 3 independent coating procedures, 1,200 force–distance curves/adhesion map (*p < 0.05).

Keratin-deficient keratinocytes show a robust activation of p38MAPK under untreated conditions (Figure 5A), which accounts for impaired Dsg3-binding forces on single molecule level (25). On the other hand, pemphigus autoantibodies were not effective to induce additional loss of cell cohesion in KtyII k.o. cells. Thus, we investigated whether autoantibody-induced p38MAPK activation is dependent on expression of keratins. Interestingly, all autoantibodies induced an activation of p38MAPK after 1 h of incubation (Figure 5A) in both KtyII wt and k.o. cell lines indicating that mechanisms leading to an activation of p38MAPK after autoantibody binding are still present in keratin-deficient keratinocytes (Figure 4A). Thus, we wondered whether inhibition of p38MAPK would restore intercellular adhesion after autoantibody treatment in both cell lines. As efficiency of p38MAPK inhibition was similar for both inhibitors SB202190 and SB203580, we used SB202190 only for the further experiments (Figure 5B). As shown above, all Ab fractions caused loss of intercellular adhesion in KtyII wt but not in KtyII k.o. cells after 24 h of incubation (Figure 5B). SB202190 in KtyII wt cells blocked loss of cohesion after autoantibody treatment and in addition restored intercellular adhesion in keratin-deficient keratinocytes to levels of wt cells (Figure 5B).

Next, we probed the effect of p38MAPK inhibition on Dsg binding properties after autoantibody incubation by AFM. Since Dsg3-binding events cannot be characterized because of autoantibody-mediated direct inhibition (11) we here focused on Dsg1-binding properties. For AFM experiments, cells were treated either with SB202190 or PF-IgG alone or in a combination using a preincubation of SB202190 for 1 h followed by addition of PF-IgG for another 1 h. After treatment cells were extensively washed and reprobed using a cantilever functionalized with Dsg1. Again, PF-IgG alone caused redistribution of Dsg1-binding events away from the cell borders as outlined above whereas no change was observed in KtyII k.o. cells (Figure 5C). SB202190 alone did not change distribution or number of Dsg1-binding events in wt keratinocytes, but led to redistribution toward the cell borders in k.o. cells (Figures 5C–E). Furthermore, SB202190 abolished PF-IgG-induced redistribution in wt cells indicating that p38MAPK signaling participates in the relocalization of Dsg1 from cell junctions in pemphigus (Figures 5C–E). Taken together, the last set of data supports the notion that loss of cell cohesion caused by both autoantibodies and, keratin deficiency is mediated or at least modulated by p38MAPK and that relocalization of Dsg1 may be a primary mechanism to destabilize keratinocyte cohesion. Moreover, together with experiments using anisomycin described earlier, the data suggest that the targets of p38MAPK autoantibodies are to some extent not dependent on expression of keratins.

## Discussion

Taken together, the data presented demonstrate that keratins differentially regulate the binding properties of Dsg1 and 3, the two major antigens of pemphigus. Moreover, we observed that direct inhibition of Dsg3 but not of Dsg1-binding occurs on living keratinocytes treated with PV-IgG and PF-IgG. We found that p38MAPK signaling is crucial for loss of cell cohesion in response to both pemphigus autoantibodies as well as keratin deficiency. Importantly, PV-IgG and PF-IgG as well as direct p38MAPK activation induced redistribution of Dsg1-binding events away from cell borders in wt keratinocytes resulting in a Dsg1 distribution pattern similar to keratin-deficient keratinocytes. No temporal sequence and thus no causative relation between keratin retraction and Dsg redistribution as the underlying mechanism for loss of intercellular adhesion are provided by the data of the study. However, the data demonstrate that keratin deficiency induces activation of p38MAPK and that p38MAPK regulates Dsg distribution. This indicates that keratin uncoupling may account for loss of cell cohesion by redistribution of Dsg1. Thus, we propose the concept that as least in part loss of intercellular adhesion in pemphigus is mediated by keratin-dependent regulation of binding properties and distribution of desmosomal cadherins *via* p38MAPK.

### Keratins Regulate Distribution and Binding Properties of Pemphigus Antigens Dsg1 and 3

Keratin filaments are crucial for mechanical properties and stability of keratinocytes (24, 41–43). Furthermore, they influence the turnover of desmosomal components (44) such as DP (45) and plakophilins (46, 47) and regulate desmosomal adhesion through signaling (22, 23, 48). Moreover, keratin filament retraction is a morphological hallmark in pemphigus pathogenesis and correlates with loss of intercellular adhesion, depletion of Dsgs from the cell membrane and is interconnected with several signaling pathways known to be crucial for pemphigus such as PKC and p38MAPK (11, 15, 25). Until now, no temporal sequence could be delineated for Dsg internalization and keratin retraction. Some studies indicate that keratin retraction does only occur when desmosomes are lost (17) whereas other studies implicate that Dsgs internalization and uncoupling from keratins are temporally closely related (Schlögl et al., this issue) or show that Dsgs which are not coupled to keratins get rapidly internalized after autoantibody treatment (49).

In the study presented here we show that keratins regulate the binding properties of Dsg1 and Dsg3, which are the major antigens in pemphigus (7, 50), and propose a new concept how keratins could directly influence desmosomal adhesion. It is conceivable that keratins by anchoring the desmosomal plaque modulate the binding properties of desmosomal cadherins which may account for the observation that retraction of keratin filaments from the desmosomal plaque after treatment with autoantibodies similar to deficiency of keratins impairs intercellular adhesion (13, 15, 22, 26). In line with this, it was reported that increased DP association to keratins strengthened intercellular adhesion and reduced autoantibody induces loss of intercellular adhesion in a pemphigus model (14, 45). With this respect, we observed the effect of keratins on desmosomal cadherin binding properties is different for Dsg isoforms. More Dsg3-binding events occurred in keratin-deficient keratinocytes whereas less interactions were detectable for Dsg1. For Dsg3, enhanced Dsg3 expression may reflect an insufficient compensatory mechanism because mRNA levels were elevated in keratin-deficient keratinocytes (25). Furthermore, it appears that keratins are not crucial for proper localization of Dsg3 at cell junctions (Figure 2C). By contrast, keratin expression was crucial for proper localization of Dsg1 to cell junctions. As known for other desmosomal cadherins this may be explained by posttranslational modifications such as phosphorylation and palmitoylation leading to altered expression or membrane localization of the respective isoform (51, 52). However, also alterations in desmosomal turnover could account for this phenomenon (9, 23, 44). Interestingly, in pemphigus patients smaller desmosomes were reported for aDsg1 but not for aDsg3 antibodies, fitting to the observation that keratins differentially regulate Dsg distribution and binding properties (53). In addition, adhesive strength of Dsg3 but not of Dsg1 binding was altered by absence of keratins (Figure 3). Thus, additionally to reduced Dsg3-binding forces keratin-dependent clustering of Dsg1 may also be crucial for strong intercellular adhesion which is well established for classical cadherins (54, 55). Changes in binding forces maybe caused by missing anchorage of the molecules or by conformational changes induced by loss of keratin coupling (42, 56) or induced by signal pathways such as p38MAPK which were shown to restore Dsg3-binding force in keratin-deficient keratinocytes (25). Finally, Dsg3 depletion was accelerated in keratin-deficient keratinocytes, which could be caused by a higher molecule mobility found when keratins are missing. Beginning of depletion of Dsg3 molecules was not detectable before 1 h after incubation with PV-IgG in wt keratinocytes which is contradictory to other studies where depletion was already detectable after 30 min of autoantibody incubation (57, 58). However, these differences in our opinion can be explained by different model systems, methodical approach and several PV-IgG fractions used in the respective studies (51, 59–61). Changes in molecule mobility may depend on PKC signaling (22, 23, 25) and be accompanied with changes in clustering of the desmosomal molecules. Thus, clustering which conforms to the note that it is crucial for proper adhesive function (5, 62), could serve as an explanation for altered forces.

### Mechanisms of Loss of Intercellular Adhesion in PF

Direct inhibition of Dsg interaction was thought to be the primary mechanism for loss of intercellular adhesion in pemphigus because autoantibodies predominantly target the EC1 domain (63). Indeed, direct inhibition of Dsg3 binding has been shown to occur after incubation with AK23 or with PV-IgG both under cell-free conditions as well as on the surface of living keratinocytes (8, 10, 11, 64). However, no direct inhibition of Dsg1 interactions was observed for both PV-IgG and PF-IgG, at least under cell-free conditions (8, 10, 12). Rather, signaling mechanisms were found to be crucial for loss of intercellular adhesion in response to pemphigus autoantibodies both in PV and PF (4, 7). Since several signaling pathways were found to be activated after binding of autoantibodies against Dsg1 and 3 such as p38MAPK, Erk, and Src (8, 37, 65–68), the molecular mechanism how PF-IgG impairs Dsg1 binding is not elucidated yet.

In this study, we demonstrate that keratins are crucial for proper localization of Dsg1 at cell junctions. Furthermore, we observed that PF-IgG led to a redistribution of Dsg1-binding events from cell junctions thereby inducing a distribution pattern similar to Dsg1 distribution of keratin-deficient keratinocytes. Together with the finding that PF-IgG similar to PV-IgG did not further impair keratinocyte cohesion in keratin-deficient cells, this suggests that uncoupling of keratin filaments from the desmosomal plaque maybe the underlying mechanism for both redistribution of Dsg1 and loss of keratinocyte cohesion. This is in line with an altered clustering of Dsg1 in pemphigus patients’ lesions which was described using electron microscopy (17, 20). Furthermore, we observed Dsg1-binding forces were reduced after 1 h of PF-IgG treatment in keratin-deficient keratinocytes but not in wt cells whereas forces were reduced in the wt monolayers after 24 h as well, suggesting that redistribution of Dsg1 may precede reduction of Dsg1-binding forces. Thus, a possible sequence of events after PF-IgG binding is conceivable in which Dsg1 redistribution caused by uncoupling from keratins and subsequently impaired Dsg1-binding forces may account for loss of intercellular adhesion. Similarly, a sequence of distinct phases has been shown for treatment with PV-IgG before (49, 69, 70). Here, depletion of non-desmosomal Dsg3 with the first 2 h was followed by rearrangement of desmosomal components into linear arrays aligned with keratin filaments between 2 and 6 h. From these arrays, desmosomal components including Dsg3 were internalized within 6–24 h, which was paralleled by disassembly of desmosomes. Furthermore, uncoupling and redistribution of Dsg3 may also be explained by the concept of Dsg non-assembly depletion hypothesis (20). Direct inhibition of Dsg3 binding occurring as fast as within 15 min (11) may facilitate depletion of both non-desmosomal as well desmosomal Dsg3. Because within the first hour of autoantibody incubation we observed redistribution of Dsg1 from cell junctions, the data of this study are in line with the hypothesis that Dsg1 after treatment with PF-IgG is affected in comparable manner, however, in absence of direct inhibition of Dsg1 interaction.

### Keratin-Dependent p38MAPK Signaling Contributes to Loss of Intercellular Adhesion in Pemphigus

Signaling pathways essentially contribute to the loss of intercellular adhesion in pemphigus (4, 7). As mentioned earlier, a broad spectrum of signaling mechanisms contribute to the loss of intercellular adhesion including PKC, Erk, Src, and p38MAPK (5). Especially, p38MAPK signaling is well characterized. Pemphigus autoantibodies induce activation of p38MAPK, and inhibition of this pathway is effective to inhibit both loss of intercellular adhesion and keratin filament alterations induced by pemphigus autoantibodies in cell culture (13, 38, 39, 71, 72). More recently, AK23- and PV-IgG-induced alterations of keratin insertion into desmosomes in human epidermis *ex vivo* as revealed by electron microscopy were shown to be dependent on p38MAPK (18). Furthermore, rapid disruption of the keratin cytoskeleton also induced p38MAPK activation whereas inhibition of p38MAPK abrogated pharmacological disruption of the keratin cytoskeleton (73–75) indicating that keratin reorganization and p38MAPK signaling are closely related.

Here, we observed that activation of p38MAPK both mediated by anisomycin as well as in response to pemphigus autoantibodies induced keratin filament retraction and led to a redistribution of Dsg3-binding events away from the cell borders in wt but not in keratin-deficient keratinocytes. Similarly, pemphigus antibodies as well anisomycin activated p38MAPK in both wt and keratin-deficient cells, the latter of which displayed robust activation of p38MAPK under resting conditions (25). Interestingly, p38MAPK activation in response to AK23 and PV-IgG was stronger in keratin-deficient cells after 1 h when compared with wt cells. All these data indicate that keratins participate in the suppression of p38MAPK activity. By contrast, anisomycin was effective to further impair cell cohesion in keratin-deficient cells whereas AK23, PV-IgG, and PF-IgG were not. Since anisomycin-induced loss of cell adhesion was abrogated by two different inhibitors of p38MAPK, loss of adhesion appears not to be due to off-target effects. Rather, this discrepancy may be explained by the observation that anisomycin is stronger to activate p38MAPK compared with autoantibodies. Alternatively, it is possible that several p38MAPK pools are available in cells (76, 77) which when activated by anisomycin contribute to loss of cell cohesion, whereas p38MAPK activation in response to autoantibodies is restricted to the pool associated with desmosomal cadherins as shown for Dsg3 (26). Indeed, immunostaining revealed that in contrast to Src only a small portion of p38MAPK was confined to cell junctions (66). Nevertheless, inhibition of p38MAPK restored intercellular adhesion after pemphigus autoantibody treatment in wt and k.o. cells and rescued keratinocyte cohesion in keratin-deficient cells indicating that p38MAPK signaling is crucial for intercellular adhesion. This is in line with former studies which show that keratin filament alteration correlate with loss of intercellular adhesion in pemphigus (11, 13, 37). Moreover, these data demonstrate that the targets of p38MAPK are in part keratin dependent, as indicated by anisomycin-mediated keratin retraction which is similarly observed after treatment with autoantibodies. In addition, given the efficiency of p38MAPK inhibitors on cell cohesion in keratin-deficient cells, keratin-independent targets must also be involved. However, it has to be noted that no temporal sequence can be ascertained by the data provided. Taken together, the data show that p38MAPK activation in response to pemphigus autoantibodies is regulated *via* keratin filaments and is critical for loss of cell adhesion. This is at least in part mediated on the level of redistribution of Dsg1 and Dsg3 molecules from cell junctions.

## Ethics Statement

The human sera used in this study were collected and used in accordance with the recommendations of ethic committee of University of Lübeck, AZ 12-178. Name of the indicated project: Autoantikörperreaktivität und Pathophysiologie bei blasenbildenden Autoimmundermatosen (Pemphigoid und Pemphigus).

## Author Contributions

FV, EW, MF, and MR performed experiments. FV, MR, and FB analyzed data. FV, TM, and VS discussed data and interpreted results. FV and JW designed the study and wrote the manuscript.

## Conflict of Interest Statement

The authors declare that the research was conducted in the absence of any commercial or financial relationships that could be construed as a potential conflict of interest.

## Supplementary Material

Figure S1 Distribution coefficient to determine localization of desmoglein binding events. **(A)** For calculation of intensity coefficient areas in close proximity to the cell borders (red rectangle) and above the nuclei (green rectangle) were chosen, intensity was measured, and a coefficient was calculated in which values <1 indicate less keratin fluorescence signal at cell border areas, thus suggesting keratin retraction under these conditions. **(B)** For calculation of distribution coefficient, areas along the cell borders (green area) and on the cell surface (red area) were marked, and respective binding frequencies were defined by calculating a ratio of number of binding events per area. Distribution coefficient was calculated as a ratio from these. Binding frequencies in which values >1 announce increased clusters of the molecules along the cell borders. **(C)** Immunostaining of KtyII wt and KtyII k.o. cells show dense filamental structures throughout the whole cell in wild-type and confirm knockout in KtyII k.o. cells. Representative of *n* = 4.

Figure S2 Dsg1-binding events in KtyII wt and k.o. cells. **(A)** Topography overview images of KtyII wt and k.o. cells. Small areas along the cell borders (green rectangles, 6 μm × 2 μm) and cell surfaces above the nucleus (red rectangles, 4 μm × 2 μm) were chosen for adhesion mapping presented in Figure 3. **(B)** Dsg1-binding frequency in KtyII wt and k.o. cells at cell border and cell surface areas under control conditions and after treatment with pemphigus foliaceus (PF)-IgG for 1 h. *n* = 3 from 3 independent coating procedures, 1,200 force–distance curves/adhesion map (**p* < 0.05). **(C,D)** Dsg1 adhesion measurements at cell borders in KtyII wt and k.o. cells under control conditions and after treatment with aDsg1 monoclonal antibody (mAb) (p124). *n* = 3 from 3 independent coating procedures, 1,200 force–distance curves/adhesion map (**p* < 0.05). **(E)** Immunostaining of keratin 14 in KtyII wt after anisomycin treatment in higher magnification for 1 h revealed keratin filament retraction. Representative images from *n* = 4.

Figure S3 p38MAPK signaling is crucial for intercellular adhesion in KtyII wt and k.o. cells. **(A)** Western blot using SB202190 and SB203580 for 2 h to inhibit and anisomycin for 1 h to activate p38MAPK. KtyII k.o. cells show activation of p38MAPK under basal conditions compared with wild-type (wt) cells. Anisomycin drastically increased p38MAPK activation, which was partially blocked by SB202190 and SB203580. For co-incubation experiments, SB202190 and SB203580 were preincubated for 1 h before anisomycin was added for 1 h. **(B)** Pictures of dissociation assay from Figure 4 confirmed impaired adhesion in keratin-deficient keratinocytes compared with wt. Activation of p38MAPK with anisomycin reduced intercellular adhesion whereas SB202190 or SB203580 improved intercellular adhesion in both cell lines (*n* > 4, **p* < 0.05).

## Abbreviations

AFM, atomic force microscopy; Dsg, desmoglein; EC, extracellular domain, PF, pemphigus foliaceus; PV, pemphigus vulgaris; NGS, normal goat serum; BSA, bovine serum albumin; PEG, polyethylenglycol; QI, quantitative imaging; FM, force mapping; FRAP, fluorescence recovery after photobleaching.
