# Supplementary figures and images for: Keratins Regulate p38MAPK-Dependent Desmoglein Binding Properties in Pemphigus
Source: Front Immunol. 2018 Mar 19;9:528. doi: 10.3389/fimmu.2018.00528 (PMC5868517; doi:10.3389/fimmu.2018.00528)

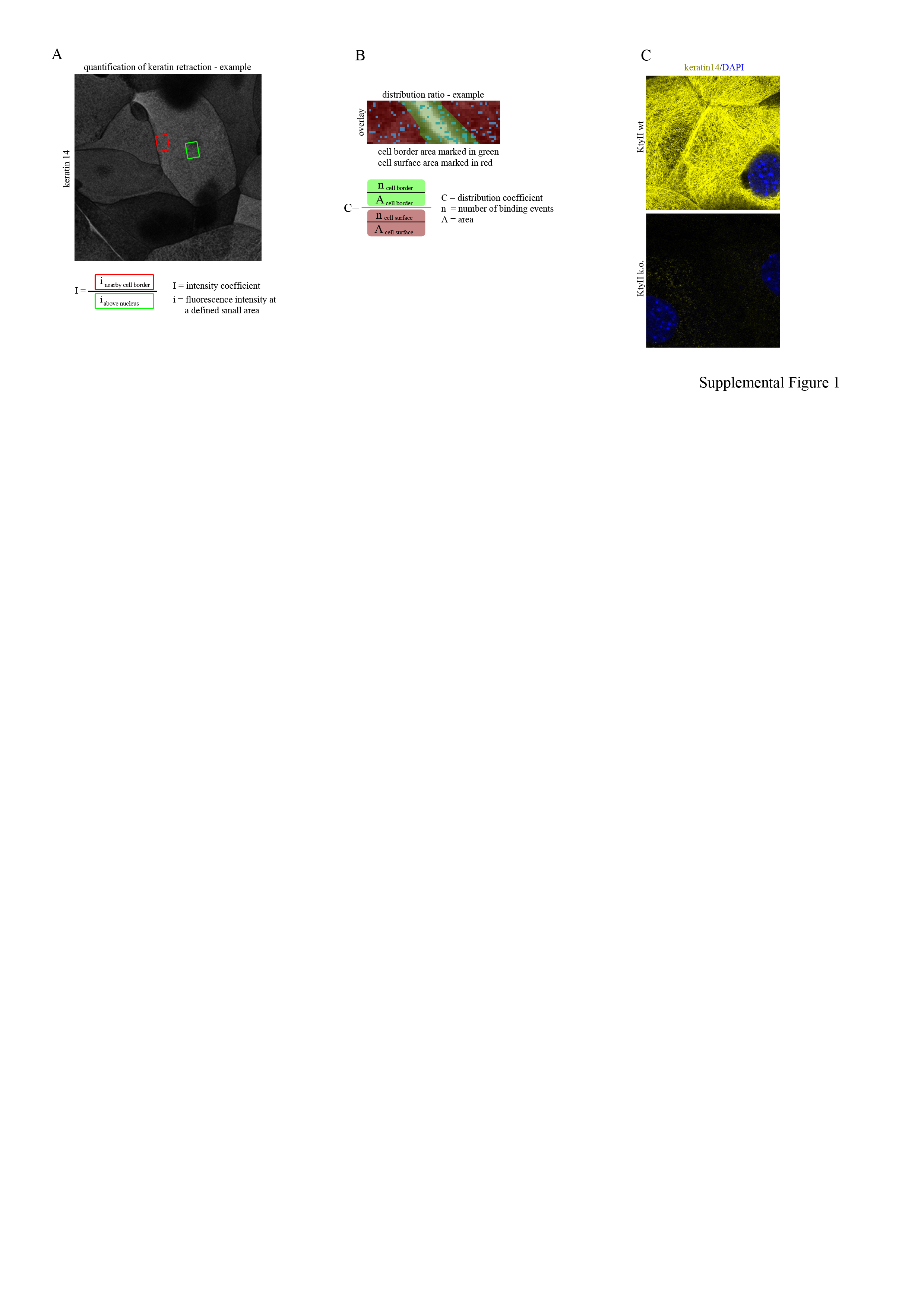

Supplement: Figure S1 — Distribution coefficient to determine localization of desmoglein binding events. (A) For calculation of intensity coefficient areas in close proximity to the cell borders (red rectangle) and above the nuclei (green rectangle) were chosen, intensity was measured, and a coefficient was calculated in which values <1 indicate less keratin fluorescence signal at cell border areas, thus suggesting keratin retraction under these conditions. (B) For calculation of distribution coefficient, areas along the cell borders (green area) and on the cell surface (red area) were marked, and respective binding frequencies were defined by calculating a ratio of number of binding events per area. Distribution coefficient was calculated as a ratio from these. Binding frequencies in which values >1 announce increased clusters of the molecules along the cell borders. (C) Immunostaining of KtyII wt and KtyII k.o. cells show dense filamental structures throughout the whole cell in wild-type and confirm knockout in KtyII k.o. cells. Representative of n = 4. [file Image_1.tif]

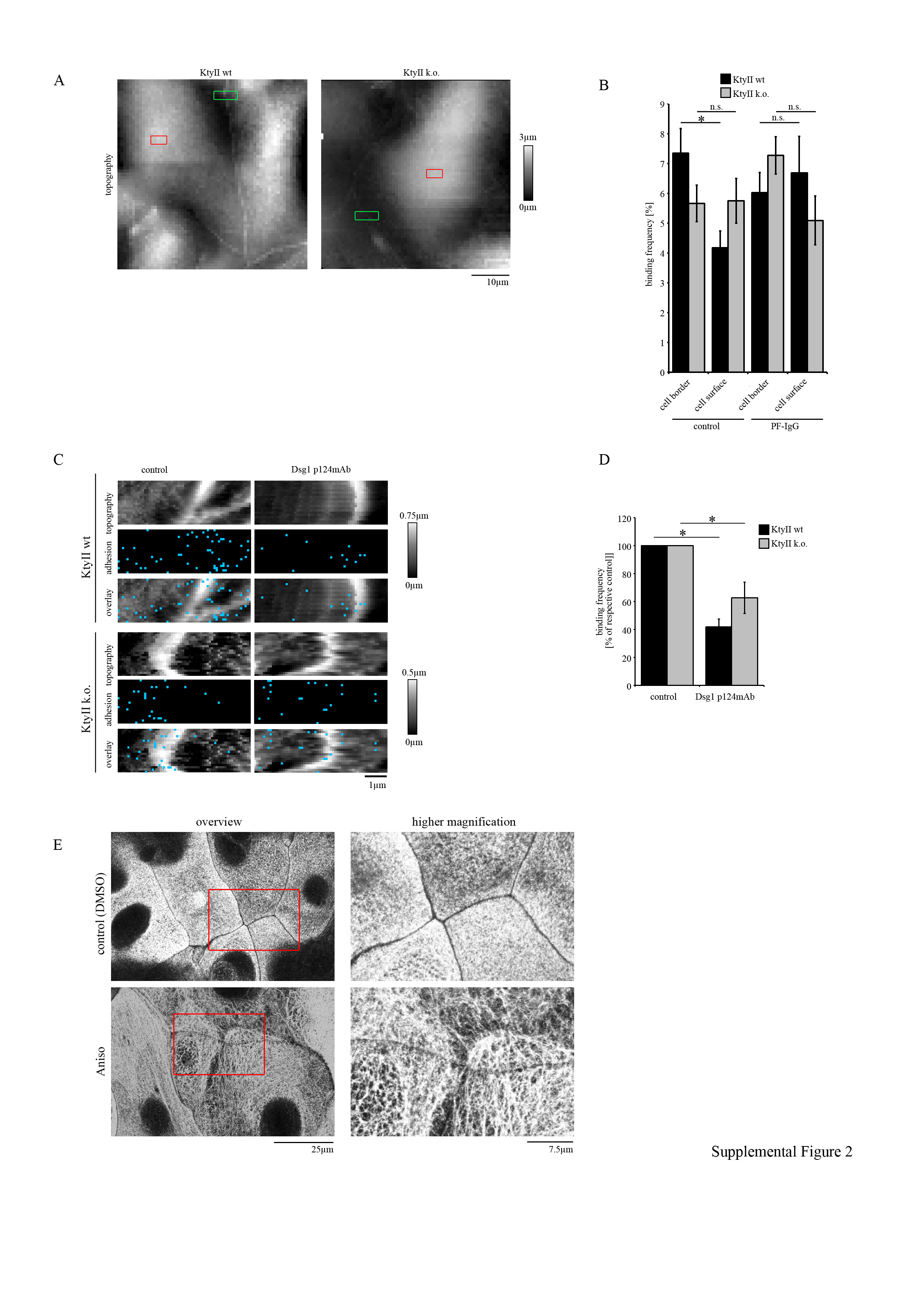

Supplement: Figure S2 — Dsg1-binding events in KtyII wt and k.o. cells. (A) Topography overview images of KtyII wt and k.o. cells. Small areas along the cell borders (green rectangles, 6 μm × 2 μm) and cell surfaces above the nucleus (red rectangles, 4 μm × 2 μm) were chosen for adhesion mapping presented in Figure 3. (B) Dsg1-binding frequency in KtyII wt and k.o. cells at cell border and cell surface areas under control conditions and after treatment with pemphigus foliaceus (PF)-IgG for 1 h. n = 3 from 3 independent coating procedures, 1,200 force–distance curves/adhesion map (*p < 0.05). (C,D) Dsg1 adhesion measurements at cell borders in KtyII wt and k.o. cells under control conditions and after treatment with aDsg1 monoclonal antibody (mAb) (p124). n = 3 from 3 independent coating procedures, 1,200 force–distance curves/adhesion map (*p < 0.05). (E) Immunostaining of keratin 14 in KtyII wt after anisomycin treatment in higher magnification for 1 h revealed keratin filament retraction. Representative images from n = 4. [file Image_2.tif]

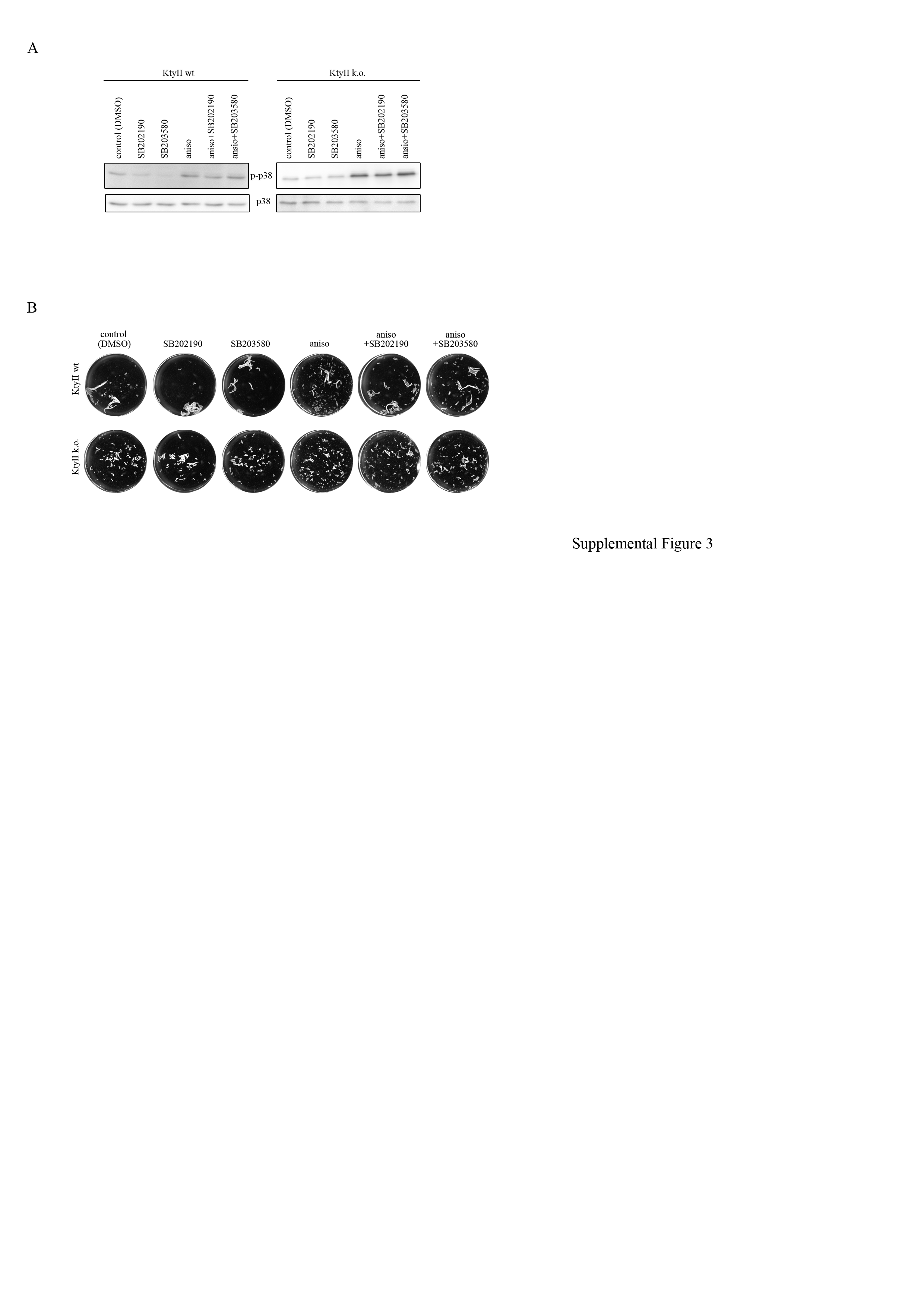

Supplement: Figure S3 — p38MAPK signaling is crucial for intercellular adhesion in KtyII wt and k.o. cells. (A) Western blot using SB202190 and SB203580 for 2 h to inhibit and anisomycin for 1 h to activate p38MAPK. KtyII k.o. cells show activation of p38MAPK under basal conditions compared with wild-type (wt) cells. Anisomycin drastically increased p38MAPK activation, which was partially blocked by SB202190 and SB203580. For co-incubation experiments, SB202190 and SB203580 were preincubated for 1 h before anisomycin was added for 1 h. (B) Pictures of dissociation assay from Figure 4 confirmed impaired adhesion in keratin-deficient keratinocytes compared with wt. Activation of p38MAPK with anisomycin reduced intercellular adhesion whereas SB202190 or SB203580 improved intercellular adhesion in both cell lines (n > 4, *p < 0.05). [file Image_3.tif]
